# Supplementary material for: Functional Recovery Caused by Human Adipose Tissue Mesenchymal Stem Cell-Derived Extracellular Vesicles Administered 24 h after Stroke in Rats
Source: Int J Mol Sci. 2021 Nov 28;22(23):12860. doi: 10.3390/ijms222312860 (PMC8657917; doi:10.3390/ijms222312860)
Supplement: Supplementary file 1 [file ijms-22-12860-s001.zip › ijms-1410220-supplementary.pdf]

International Journal of
Molecular Sciences

MDPI

Article

Functional recovery caused by human adipose tissue mesenchymal stem cell-derived extracellular vesicles administered 24 hours after stroke in rats

Francieli Rohden [1,2,*], Luciele Varaschini Teixeira [1,2], Luis Pedro Bernardi [1,3], Pamela Cristina Lukasewicz Ferreira [1], Mariana Colombo [4], Geciele Rodrigues Teixeira [5], Fernanda dos Santos de Oliveira [5], Elizabeth Obino Cirne Lima [5], Fátima Costa Rodrigues Guma [1] and Diogo Onofre Souza [1,*]

Supplementary Figure S1. EVs (200 μg/kg) effect on individual performance of animals.

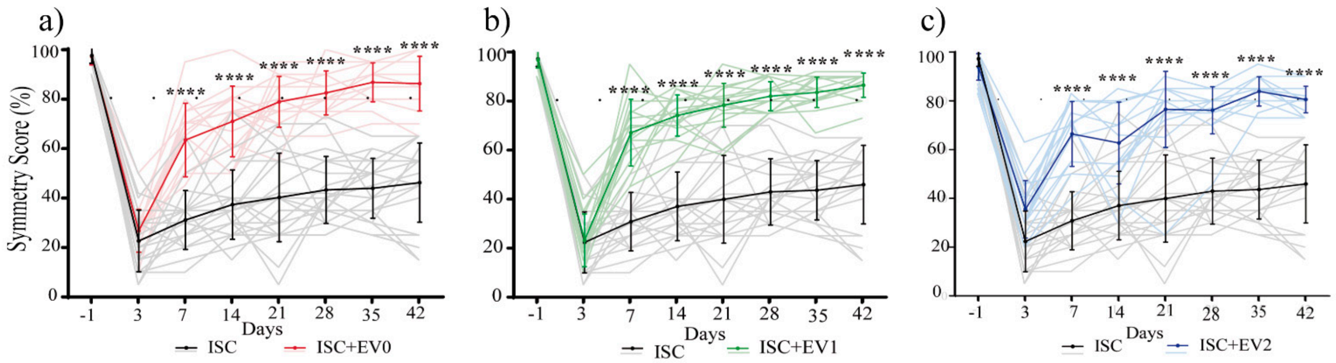

Figure S1. (a-c) EVs (200 μg/kg) effect, represented as mean ± SD (strong lines) and individual performance of each animal (soft lines). **(a)** ISC + EV0 (n=17), **(b)** ISC + EV1 (n=17) and **(c)** ISC + EV2 (n=17). ISC (n=22). Data were analyzed by two-way ANOVA followed by Tukey's multiple comparisons test; ****$p < 0.0001$, compared to ISC group.

Supplementary Figure S2: Brain blood vessels parameters in SS region

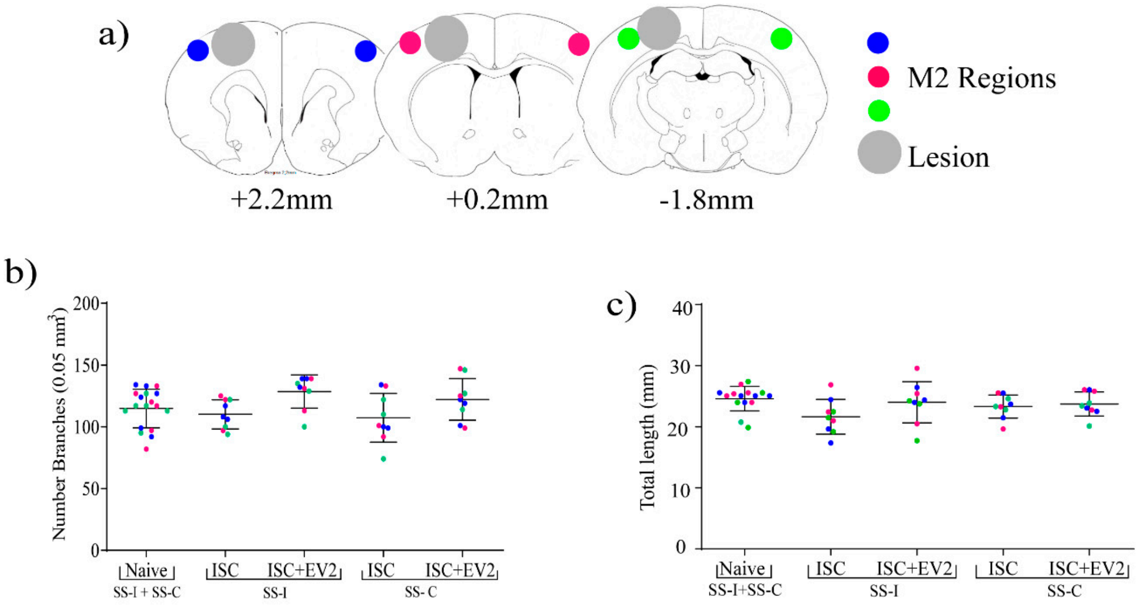

Figure S2. Brain blood vessels parameters. The parameters were measured on somatosensory regions of the cortex (SS) in the naive group and in the group treated with EV2 at day 42 after treatment; volume of piece: 0.05 mm^3. **(a)** Schematic representation of the brain positions analyzed (+2.20 mm, +0.2 mm and −1.88 mm to Bregma) in peri-infarct region and its contralateral equivalent. **(b)** Number of vessels branches in SS-Ipsilateral (SS-I) and SS-Contralateral (SS-C) regions; **(c)** Total vessels lengths in SS-I and SS-C regions. Statistical analysis using the unpaired t-test. Data are reported as the mean ± SD. (n=3). No significant difference was observed among groups.
